# Identification of OLED Degradation Scenarios by Kinetic Monte Carlo Simulations of Lifetime Experiments

**DOI:** 10.3389/fchem.2021.823210

**Published:** 2022-01-27

**Authors:** Christoph Hauenstein, Stefano Gottardi, Engin Torun, Reinder Coehoorn, Harm van Eersel

**Affiliations:** ^1^ Simbeyond B.V., Eindhoven, Netherlands; ^2^ Department of Applied Physics, Eindhoven University of Technology, Eindhoven, Netherlands; ^3^ Institute for Complex Molecular Systems, Eindhoven University of Technology, Eindhoven, Netherlands

**Keywords:** TADF, OLED (organic light emitting diodes), degradation, kinetic Monte Carlo (KMC), accelerated lifetime measurements, OLED degradation, OLED lifetime

## Abstract

We show how three-dimensions kinetic Monte Carlo simulations can be used to carry out an operational lifetime study of thermally activated delayed fluorescence (TADF) organic light-emitting diodes (OLEDs) and to deduce the sensitivity to various degradation scenarios. The approach is demonstrated for an experimentally well-characterized efficient green-emitting device. The simulation workflow includes an equilibration phase, an equilibrated pristine state phase and a degradation phase. Acceleration of the simulations by extrapolation from simulations at large current densities makes the simulation time realistically feasible. Such a procedure is also often followed in experimental studies. Degradation is assumed to be triggered by exciton-polaron quenching and exciton-exciton annihilation processes. A comparison of the simulated and experimental time-dependence of the luminance decay provides the probability that a degradation-triggering event leads to the formation of a degraded molecule. For the TADF OLED that has been studied, this parameter is only weakly dependent on the assumed scenario, provided that the degraded molecules are assumed to form trap sites, and is found to be $∼\left(0.2-0.7\right)\times 10^{-9}$ . The approach is expected to enable systematic in silico studies of the operational lifetime and its sensitivity to the material composition, layer structure, charge carrier balance, and the use of refined device principles such as hyperfluorescence.

## 1 Introduction

In spite of impressive developments in the past decades, the range of applications of organic light-emitting diodes (OLEDs) could still be widened significantly by improving their stability under operational conditions. Most research efforts are focused on blue OLEDs, which are most sensitive to lifetime-limiting degradation processes (Lee et al., 2019). Phenomenological lifetime studies focus on measurements of the luminance decay and the voltage shift over time as a function of the operational conditions, such as the current density and the temperature. However, it is generally not obvious how from such measurements the microscopic mechanism that causes the observed changes over lifetime can be deduced, where in the device the degradation occurs, what degradation product is formed, and which fraction of the material has degraded. As a result, it is often not possible to deduce directly from degradation measurements what changes of the material composition or layer stack architecture could lead to an improved lifetime. The impact of degradation on charge or exciton dynamics can sometimes be recognized by experimental observations, for example by the appearance of exciplex emission in Tanaka et al. (2019), but even then only qualitatively. As an alternative to such an experimental top-down approach, bottom-up approaches that aim at predicting the probability of degradation processes, the nature of the degradation products and their electronic and optical properties from (combined) experimental and quantum chemistry studies are underway (Kim et al., 2020). However, such studies have yet to be experimentally validated. Furthermore, the understanding of degradation at the molecular scale does not yet directly provide understanding of the complex interplay of all transport and excitonic processes that together determine the observed lifetime.

In this paper, we explore the prospects of a third route, which is based on simulations including a mechanistic description of degradation processes at the molecular scale. The simulations allow us to predict the results of lifetime experiments, depending on the chosen descriptions of the device degradation in the simulations. We compare several such descriptions, which we call “degradation scenarios.” By comparing different scenarios we investigate to what extent they can be distinguished and to what extent a comparison with the experimental data allows identifying more likely scenarios. We use for that purpose three-dimensional kinetic Monte Carlo (KMC) simulations (Bässler, 1993; Mesta et al., 2016). Our approach to KMC simulations of device degradation was introduced and applied to model OLED devices by Coehoorn et al. (2015) and Kordt et al. (2017), and was applied to a phosphorescent OLED for one specific simulation scenario (exciton polaron-quenching triggered degradation, followed by the formation of deep traps) by Shen and Giebink (2015). Here, we focus on a green thermally activated delayed fluorescence (TADF) OLEDs for which an extensive set of experimental initial state (*t* = 0, where *t* is the time) and *t*-dependent data is available (Furukawa et al., 2015), and for which also a successful KMC simulation study of the pristine device has already been done (Gottardi et al., 2019). We study for several degradation scenarios whether these could explain the observed luminance decay, up to the experimental LT50 lifetime of about 1,470 h, and the observed time-dependent voltage increase, which is about 0.8 V at the LT50 lifetime. The LT50 lifetime is the time at which the luminance of the device has decayed to half of its initial value. The observation of such a voltage increase is quite common, and indicates that upon degradation trap sites are formed or that an injection barrier at an interface is increased (Kondakov, 2008; Scholz et al., 2015). Exciton-polaron quenching and exciton-exciton annihilation are in all scenarios assumed to be the degradation-triggering processes. The scenarios differ concerning the assumed location (limited to the emitter material or affecting all materials) and the properties of the resulting degradation product. Degradation results in material products with modified electronic and excitonic energy levels (“inactive” or trapping) and modified photophysical rates.

The paper is structured as follows. First, in Section 2, an overview is given of the device characteristic of the pristine OLED, before degradation, obtained from KMC simulations. This section first gives a brief summary of the KMC method (Section 2.1) and an overview of the materials and layer structure of the TADF OLED that is studied. We then analyze the experimental and simulated current density versus voltage (*J*(*V*)) curves, the external quantum efficiency as a function of *J* (roll-off curve) and the hole, electron, singlet and triplet exciton density profiles. In the second part of the paper (Section 3), the degradation of the device is investigated for six different degradation scenarios. In Section 3.1, we describe how degradation is implemented in the KMC simulations. Section 3.2 gives an overview of the considered degradation scenarios and degradation products. The experimental and simulated time-dependent luminance decay and voltage shift and the degradation profiles in the device are presented in Section 3.3. Finally, Section 4 contains a summary of the results and conclusions, and an outlook on extensions of these degradation simulation studies.

## 2 Characterization of the Pristine State of the Device

**FIGURE 1:**
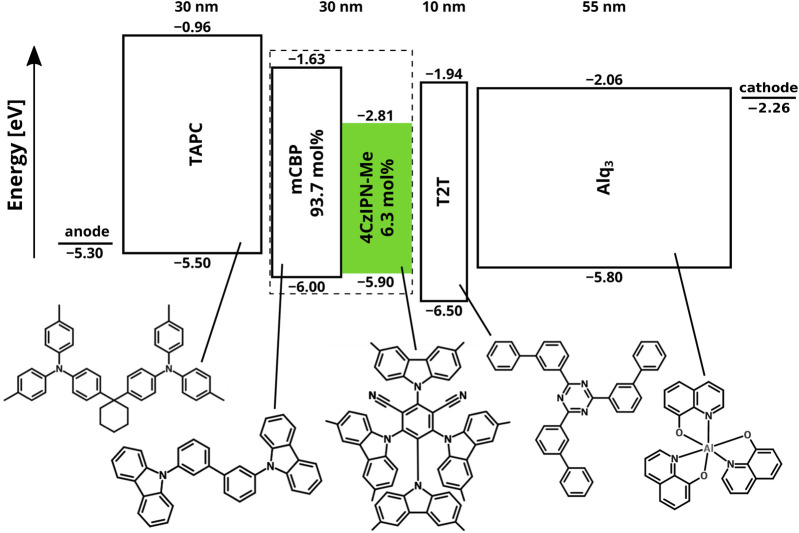
Layer structure and energy levels (with respect to the vacuum level, in units eV) of the TADF device studied in this paper. The HOMO energies, *E*_HOMO_, were determined by photoelectron yield spectroscopy (Nakanotani et al., 2014; Furukawa et al., 2015). The LUMO energies, *E*_LUMO_, are obtained using *E*_LUMO_ = *E*_HOMO_ + *E*_S_ + *E*_S,b_, with *E*_S_ the singlet energy (see Table 1) and *E*_S,b_ the singlet binding energy. The importance of including the proper value for the exciton binding energy in the value of the LUMO that is used in KMC simulations was demonstrated in Coehoorn et al. (2015) and Ligthart et al. (2021). Based on inverse photoelefctron spectroscopy, the values of *E*_S,b_ for 4CzIPN-Me and Alq_3_ were taken as 0.61 and 1.21 eV, respectively. ^1^The exciton binding energy of 4CzIPN-Me is assumed to be equal to that of 4CzIPN, studied in Yoshida and Yoshizaki (2015). For the other materials we take *E*_S,b_ = 1.0 eV (Knupfer, 2003). The injection barriers at the anode, with a hole injection layer [see Furukawa et al. (2015)], and at the LiF-Al cathode layer are taken equal to 0.2 eV.

Figure 1 shows the layer stack and the energy level structure of the TADF device studied in this work. The emissive layer (EML) consists of the host material 3,3-di(9*H*-carbazol-9-yl)biphenyl (mCBP) and 6.3 mol% of the TADF material (2s,4r,6s)–2,4,5,6-tetrakis(3,6-dimethyl-9*H*-carbazol-9-yl)isophthalonitrile (4CzIPN-Me), which is highlighted in green. The hole transport (HT), hole blocking (HB), and electron transport (ET) layers are composed of 4,4′-cyclohexylidenebis[*N*,*N*-bis(4-methylphenyl)benzenamine] (TAPC), 2,4,6-tris(biphenyl-3-yl)-1,3,5-triazine (T2T), and tris(8-hydroxyquinolinato)aluminum (Alq_3_), respectively. This OLED structure was studied experimentally by Furukawa et al. (2015), and using KMC simulations by Gottardi et al. (2019). In this section, we briefly summarize the simulation approach and the results obtained for the pristine device state, i.e., before degradation, with a focus on additional results that were not included in Gottardi et al. (2019) but are relevant to gaining understanding of the effects of degradation processes.

### 2.1 Method: Kinetic Monte Carlo Simulations

The KMC simulations are performed with the software tool Bumblebee^2^ (van Eersel et al., 2014; Coehoorn et al., 2015). The method that is used to describe charge injection, charge transfer and excitonic processes is the same as was employed in earlier work on phosphorescent OLEDs (van Eersel et al., 2014) and in the earlier work on the TADF OLED that is studied here (Gottardi et al., 2019). In the latter study, it was found that a good agreement with the experimental current-voltage (*J*(*V*)) curve and the experimental roll-off of the external quantum efficiency (EQE, *η*_EQE_) could be obtained. The set of simulation parameters that was used has also enabled successful modeling of other devices in earlier work (Mesta et al., 2013). Below is a description of these settings, which are also summarized in Supplementary Table S1.

The stack diagram in Figure 1 gives the frontier orbital energy level structure, including a brief motivation in the caption. The molecular sites reside on a simple cubic grid with a lattice spacing of 1 nm, a typical average intermolecular distance. The intermolecular transfer of charge carriers (hopping) is described using the Miller-Abrahams (MA) formalism (Miller and Abrahams, 1960), with an assumed wave function decay length of *λ* = 0.3 nm. The nearest-neighbor hopping attempt rate is taken to be *ν*_1,h_ = 1.66 × 10^10^ s^−1^ for holes and *ν*_1,e_ = 0.166 × 10^10^ s^−1^ for electrons. These hopping rates give typical mobilities for these types of materials when simulating a time-of-flight measurement [see SM of Gottardi et al. (2019)]. The tenfold reduced electron hopping rate mimics the effect of electron traps which are often found in organic small-molecule materials (Mesta et al., 2013; Kotadiya et al., 2019). The energetic disorder of the highest occupied molecular orbital (HOMO) and lowest unoccupied molecular orbital (LUMO) energies is included by assuming a spatially uncorrelated Gaussian density of states (DOS) with a standard deviation of *σ* = 0.1 eV. Exciton generation is described as hopping of a charge carrier to a site where an oppositely charged polaron is already located, but taking the exciton binding energy into account (van Eersel et al., 2014). The ratio of the formation of singlets and triplets is assumed to be equal to the spin statistical value of 1:3. Exciton dissociation is treated analogously to generation. A Gaussian density of states with a standard deviation of *σ* = 0.05 eV is used for modeling the energetic disorder of the singlet and triplet energies.

The radiative and non-radiative decay rates of the excitons and the (reverse) intersystem crossing rates for 4CzIPN-Me are taken from Furukawa et al. (2015), or are estimated in the case of their absence. Material-specific parameters of the stack used in the KMC simulations are provided in Table 1. The diffusion of excitons is described as a result of MA-type Dexter processes, with an attempt rate to a nearest-neighbor of 2.1 × 10^7^ s^−1^, plus (in the case of singlets) Förster-type transfer, with a Förster radius of 1.5 nm. The rates include a Boltzmann factor that accounts for the exciton energy differences between the sites. The resulting reduction of the transfer rate to molecules with a higher exciton energy mimics the effect of the reduced overlap between the absorption and emission spectra. Similarly, exciton-polaron quenching and exciton-exciton annihilation are described as a result of a Dexter-type transfer process, with a rate that is equal to the hopping attempt frequency to a site on which no polaron or exciton is present (see above), plus (in the case of singlets) a rate for Förster-type processes. The Förster radius for quenching and annihilation is taken to be 3.5 nm, which can realistically describe these processes in phosphorescent OLEDs (Mesta et al., 2016; Ligthart et al., 2018, 2021). Upon an exciton-polaron quenching process, the exciton is assumed to be transferred onto the site of a polaron and is lost. Upon an SSA, STA, TSA, or TTA annihilation (A) processes (S = singlet and T = triplet), the first exciton is assumed to move onto the second exciton and is annihilated in the process. The remaining exciton will keep its original spin, with the exception of TSA that results in a triplet (such that the overall spin is conserved) and TTA which has a 25% chance to result in a singlet.

**TABLE 1:** Material-specific parameters used for the KMC simulations. *E*_HOMO_ and *E*_LUMO_ are given with respect to the vacuum level. The singlet (triplet) energies *E*_S(T)_ and the radiative (non-radiative) decay rates *k*_rad_ (*k*_nr_) are obtained from the references indicated or are given an assumed value. Values are taken from the following papers: [1] Nakanotani et al. (2014), [2] Jankus et al. (2014), [3] Seino et al. (2014), [4] Hensel and Bässler (1992), [5] Furukawa et al. (2015), [6] Yoshida and Yoshizaki (2015), [7] Chen et al. (2009), [8] Burrows et al. (2003), and [9] Matsumoto et al. (2008).

Material	*E*_HOMO_	*E*_LUMO_	*E*_S_	*E*_T_	*k*_rad,S_ (*k*_nr,S_)	*k*_rad,T_ (*k*_nr,T_)
	[eV]	[eV]	[eV]	[eV]	[10^6^ s^−1^]	[10^6^ s^−1^]
TAPC	−5.50 [1]	−0.96	3.54 [2]	2.95 [2]	8 (92) [3,4]	0 (0.01)
mCBP	−6.00 [5]	−1.63	3.37 [1]	2.90 [1]	100 (100)	0 (0.01)
4CzIPN-Me	−5.90 [5,6]	−2.81 [6]	2.48 [5,6]	2.43 [5,6]	25 (0) [5]	0 (0.2) [5]
T2T	−6.50 [5]	−1.94	3.56 [7]	2.8 [7]	100 (100)	0 (0.01)
Alq_3_	−5.80 [6]	−2.06 [6]	2.53 [6]	2.1 [8]	16.7 (83.3) [9]	0 (0.01)

All device simulations were carried out for several boxes, each with a different disorder configuration, spanning at least 50 × 50 nm^2^ up to 100 × 100 nm^2^, and the results are averages over 5 to 10 (for pristine device simulations) or at least 32 (for degradation simulations) of such boxes.

The simulation settings outlined above are in two aspects different from those in the earlier KMC study by the finding Gottardi et al. (2019). Firstly, 50% reduced values of the first nearest neighbor hopping attempt rate for holes and electrons have been used, motivated by the finding by Gottardi et al. (2019) that the current density is a factor of two too high. With this small adaptation the *J*(*V*) characteristics indeed agree excellently with experiment while the effect on the excitonic characteristics such as the *η*_EQE_(*J*) (roll-off) curve is small, as shown in Supplementary Figure S1. Secondly, in the work of Gottardi et al. (2019) only Förster-type processes between emitter sites were considered. That may be expected to be a good approximation when considering Förster-type exciton diffusion, because the singlet energy of the emitter is well below the absorption edge for the neutral ground state of all other materials in the layer stack. However, we cannot exclude that there is considerable spectral overlap of the emitter singlet spectrum and the absorption spectrum of one or more of the other materials when these are occupied by polarons or excitons. We therefore extend in this study our description of Förster-type processes, such that also between other materials (in the EML as well as in the transport layers) Förster-type quenching and annihilation processes are included with a Förster radius of 3.5 nm.

### 2.2 Simulation Results

**FIGURE 2:**
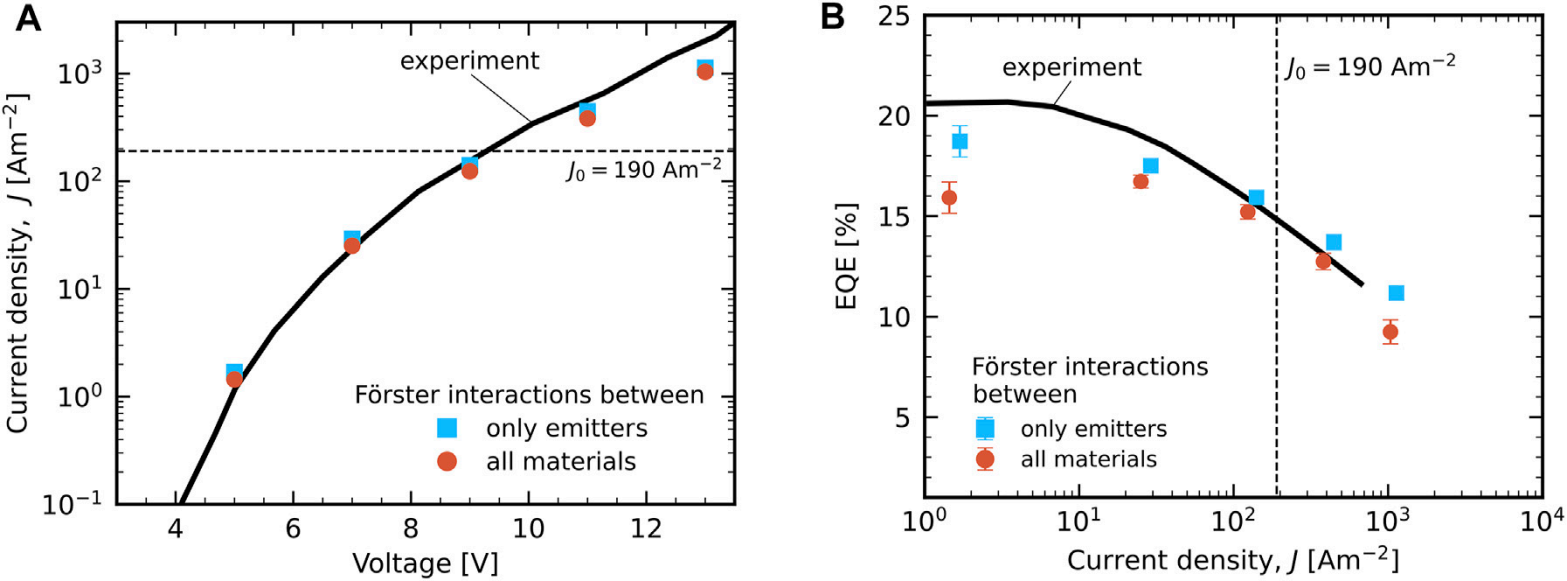
Experimental (full black curves; from Furukawa et al. (2015)) and KMC simulation results of the device in its pristine state, considering Förster-type processes only between sites of the emitter material (blue squares) or between all materials (red spheres). **(A)** *J*(*V*) characteristics. **(B)** the EQE roll-off *η*_EQE_(*J*), assuming a light outcoupling efficiency of 25%. In both panels the current density at which the degradation simulations are performed, *J*_0_ = 190 Am^−2^, is indicated by a dashed line.

Figure 2A shows the simulated and experimental *J*(*V*) characteristics of the pristine device. Simulations for two descriptions of the Förster processes are included. Dexter-mediated diffusion, quenching and annihilation are included in both cases and among all the materials. In the “emitter-only” case (blue squares) we consider Förster-type processes (diffusion, quenching and annihilation) only between sites of the emitter material. In the “all materials” case (red spheres) we consider the possibility of Förster-type processes between all materials. Because the two cases impact only the exciton dynamics, the change in current density is found to be extremely small. For both of them the simulation results match very well with experiment.

Figure 2B shows the simulated and experimental results for the EQE roll-off, *η*_EQE_(*J*), assuming a value of 25% for the light outcoupling efficiency. In Furukawa et al. (2015), this value is estimated to be in the range of 20–30%. In the absence of optical modelling the light outcoupling efficiency presents the largest uncertainty for the comparison of the EQE between simulation and experiment. When including Förster-type processes between all materials the EQE is overall somewhat reduced as compared to the “emitter-only” case, in particular at the smallest voltage (5 V) and current density considered. It falls then close to the upper edge of the estimated outcoupling efficiency, which would even have to be larger than 30% to describe the experimental EQE at low current densities. We therefore consider the case that includes Förster-type bimolecular interactions of excitons on the emitters with polarons and excitons on all materials as the strongest loss-inducing setting that we can reasonably consider. In the following section we will show where these losses occur and consequently where the degradation is expected to be strongest. Unless mentioned otherwise, all simulation data shown throughout the paper use the more extensive “all materials” Förster setting and are carried out for *J*_0_ = 190 Am^−2^ (dashed lines in Figures 2A,B).

**FIGURE 3:**
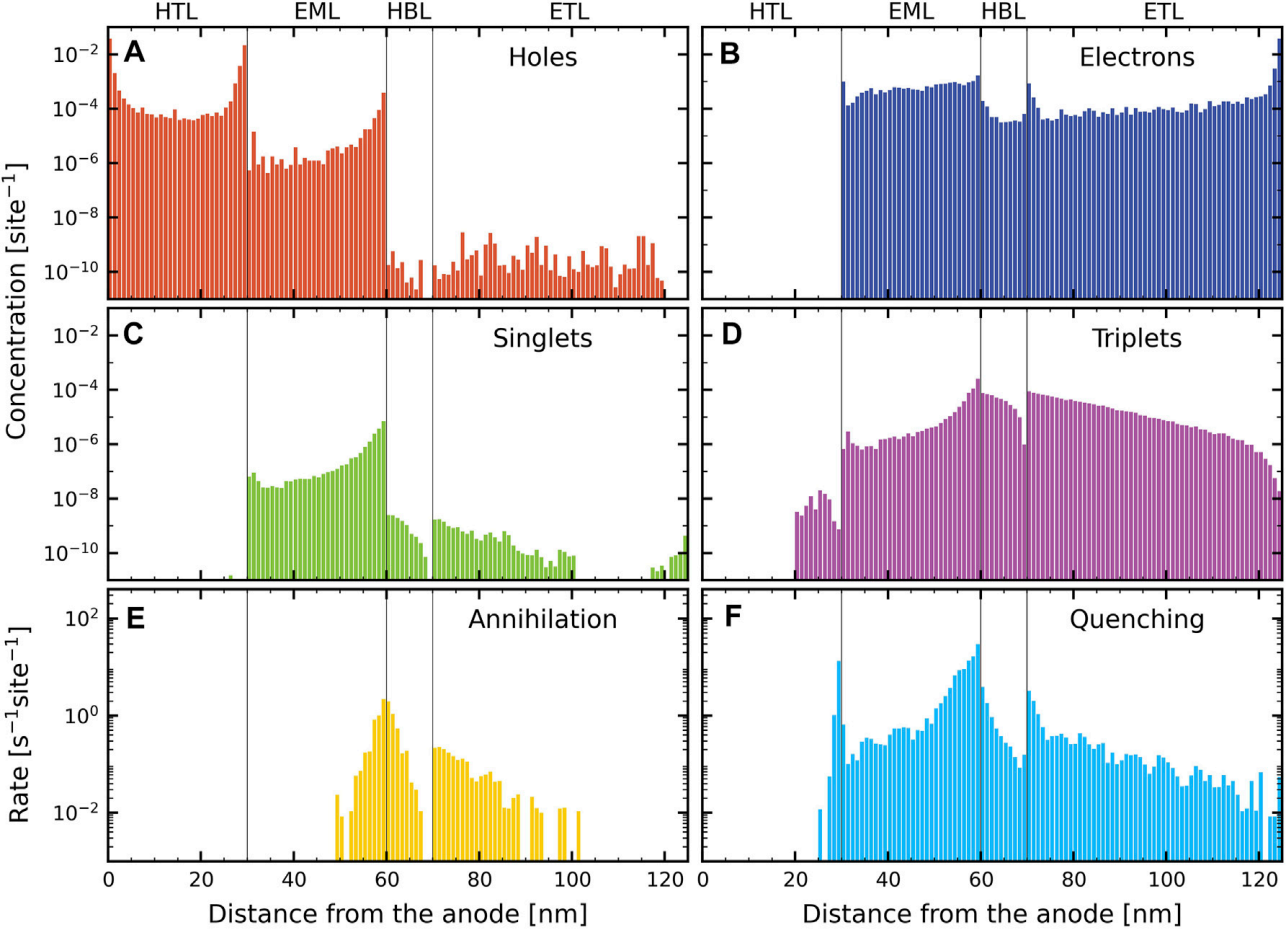
Layer-resolved profiles, obtained from simulations of the pristine device and with Förster processes between all materials, at 9 V. **(A)** Hole concentration, **(B)** electron concentration, **(C)** singlet concentration, **(D)** triplet concentration, **(E)** annihilation rate (total for S–S, S–T, T–S and T–T annihilations), and **(F)** quenching rate (total for singlets and triplets with holes and electrons).

We analyze the device simulation results at 9 V in Figure 3, for which the current density is close to *J*_0_. Panels (A) and (B) show the hole and electron concentration profiles, respectively. Panel (A) shows that the relatively large injection barrier for holes at the HTL∣EML interface gives rise to substantial hole accumulation at that interface. However, holes in the EML are relatively mobile, as the difference between the HOMO energies of the host and TADF-guest molecules is very small. As a result, the hole concentration is strongly asymmetric, and largest near the cathode-side of the EML, where they are quite effectively blocked by the HBL. Panel (B) shows that there is no electron accumulation at the T2T side of the interface with the EML, as may be understood from the absence of an electron barrier from the T2T layer to the emitter in the EML. In the EML, the electrons are less mobile than the holes, as a result of extremely strong trapping on the emitter sites (see Figure 1), though at concentrations of 6.3 mol% some charge transport via the guest is expected to occur. The overall result of these effects is a relatively weak gradient of the electron density across the EML. Panel (B) also shows that at this voltage, perfect electron blocking occurs at the TAPC hole transport layer. Panels (C) and (D) show the singlet and triplet exciton profiles. The singlet profile in each layer is proportional to the emission profile in that layer. As expected from the hole and electron concentration profiles, the device is unbalanced towards the cathode-side of the EML, with a large fraction of the emission occurring close to the HBL. Panel (D) shows that there is significant triplet deconfinement from the EML to the HTL and the HBL. These excitons outside of the EML are finally lost to non-radiative decay (predominantly triplets) and bimolecular processes (predominantly singlets). Panels (E) and (F) show the quenching rate and annihilation rate profiles, respectively. Most bimolecular losses occur in the EML, with a strong imbalance towards the HBL interface. Even though there is no strong electron accumulation at the HBL-side of the HBL∣EML interface, the high exciton density at the EML side of this interface gives rise to a significant amount of quenching in the HBL. Similarly, the high density of accumulated holes at the HTL-side of the HTL∣EML interface leads to strong singlet-hole quenching at that interface.

**FIGURE 4:**
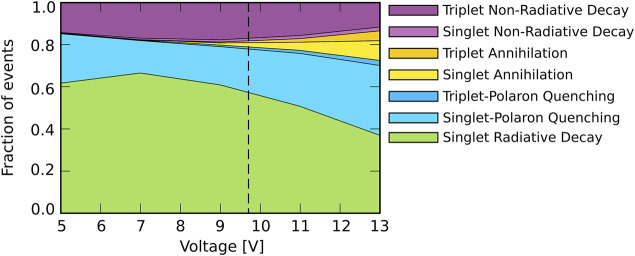
Relative contribution of the various excitonic processes for singlets and triplets with Förster processes between all materials. The dashed vertical line represents the voltage that corresponds to *J*_0_ = 190 Am^−2^ at which the degradation simulations are performed.

Figure 4 shows for the simulations with Förster processes between all materials the voltage dependence of the relative contributions of the various excitonic processes. A dashed vertical line in the figure indicates the voltage that corresponds to *J*_0_ = 190 Am^−2^ for which the degradation simulations are performed. The figure shows that at all voltages the losses are predominantly due to singlet-polaron quenching. The singlet excitons therefore undergo more quenching than the triplet excitons despite a lower overall density, which is a result of the high Förster-mediated interaction rates as compared to the Dexter-mediated rates. At low voltages, the non-radiative decay of triplets is the only other source of losses. At the selected voltage the role of annihilation is still small, though it increases at higher voltages. As shown in Supplementary Figure S2 a similar result is obtained for the “emitter-only” scenario. A slightly reduced polaron quenching contribution results in a slightly higher overall EQE.

## 3 Device Degradation

In this section we first describe how degradation is implemented in the KMC simulations (Section 3.1). In Section 3.2 the four different degradation scenarios that are investigated in this work are introduced. In Section 3.3 the simulations results for these scenarios are compared with the experimental results from Furukawa et al. (2015).

### 3.1 KMC Simulations of Device Lifetime

#### 3.1.1 Degradation Events in KMC Simulations

Degradation is implemented in the KMC simulations by 1) defining one or more processes (KMC events) that trigger degradation, 2) defining for each process a degradation probability *P*_deg_, and 3) defining the degradation product (the properties of the degraded molecule). A degradation scenario refers to a simulation that contains a specific set of such degradation triggering processes and their products. To compare different degradation scenarios we perform complete device simulations that differ in one or several aspects of these degradation parameters, but have otherwise identical settings. Any type of KMC event can be chosen as a degradation trigger, and each material can also have its own degradation triggers and products. Possible examples of such triggering events are a hop of an electron or hole to a molecule (“monomolecular degradation”) or an exciton-polaron quenching event (“bimolecular degradation”) (Giebink et al., 2008; Scholz et al., 2015). When such a triggering event occurs, the molecule on which the event takes place is with a probability *P*_deg_ converted into a degraded molecule with a predefined set of different properties. Once a site has been degraded, it keeps its degraded properties for the rest of the simulation. In typical degradation simulations, the degraded molecules have a reduced radiative decay rate (or are taken to be non-emissive). OLED lifetime studies often show a change of the voltage when operating the device at constant current density. In order to mimic such an effect, a change of the HOMO and/or LUMO energy of the degraded molecules can be assumed, so that they form a hole or electron trap.

#### 3.1.2 Simulation Phases and Lifetime Simulation Strategy

In KMC OLED lifetime simulations the total simulated time may be viewed as consisting of three successive phases: the equilibration phase, the equilibrated (pristine) phase and the degradation phase. In practice, the simulations usually start with an empty box, i.e., without electrons and holes. The simulations are most conveniently performed by imposing a fixed bias voltage *V*. This procedure has been employed in all our previous work. During the equilibration phase, the device is filled with injected electrons and holes, leading to a current density that is shortly after the start of the simulations relatively large, and a radiative decay rate that increases with time. In other words, the device is “turned on.” It is often not well possible to strictly define the time at which this equilibration process has ended and the dynamic equilibrium has been established. We usually regard the time at which the current density has converged to within one or a few percent of the long-time average as a proper value.

**FIGURE 5:**
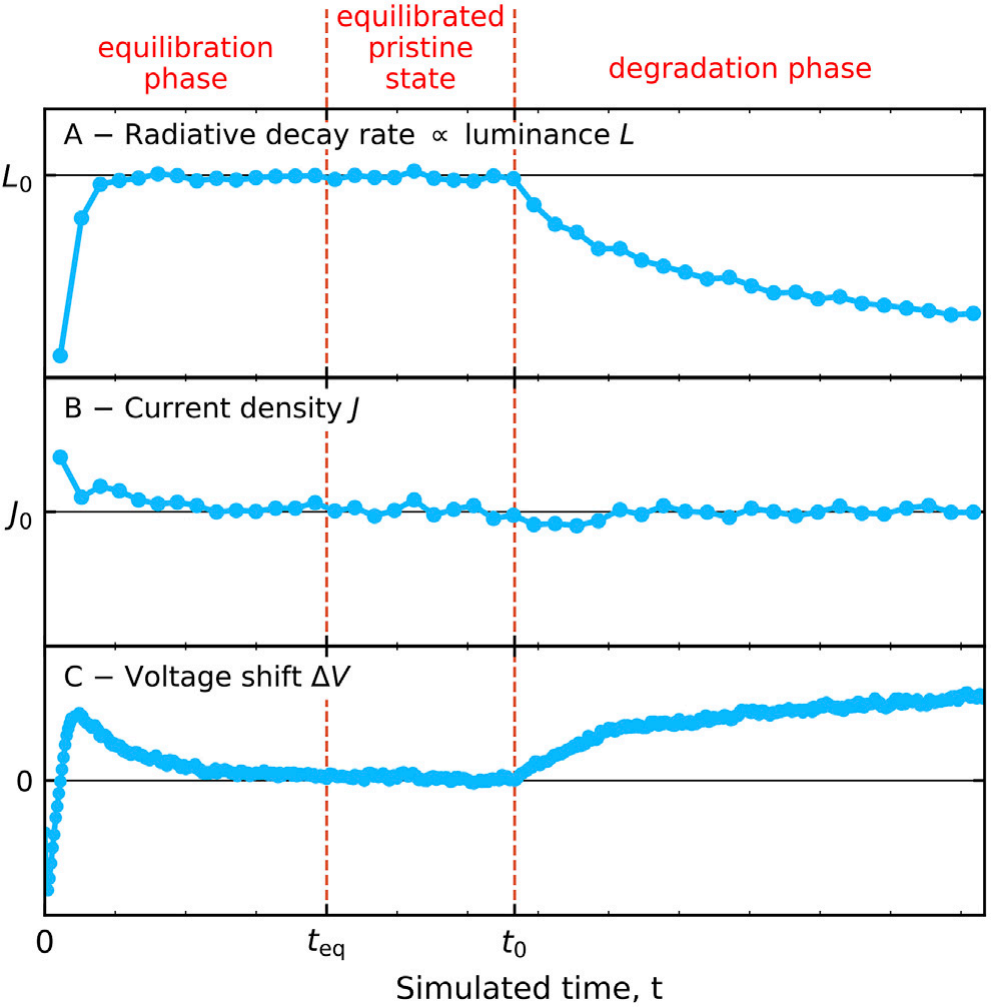
Schematic view of the three phases that can be distinguished when carrying out a KMC degradation simulation using a constant-current mode. Degradation is switched off during the equilibration phase, where the device is brought to a dynamic equilibrium condition, and during a phase that starts at a time *t*_eq_ within which the device is in the equilibrated pristine state and within which the device performance is initially determined. The degradation phase begins at a time *t*_0_ at which the degradation processes are enabled. **(A)** Radiative decay rate *R*(*t*), which is proportional to the luminance *L*(*t*). **(B)** Actual current density *J*(*t*), which is kept close to the desired current density *J*_0_ by a feedback loop that determines the applied voltage. **(C)** Shift of the applied voltage Δ*V*(*t*) with respect to the pristine state voltage.

OLED lifetime experiments are often carried out for a fixed current density, because such a driving principle is generally used in applications. The OLED lifetime studies on which we report in this paper have therefore also been carried out under constant-current conditions. For that purpose, a target current density *J*_0_ is defined at the start of the simulation, and an estimate of the voltage that will be required to maintain this current density is applied during a short predefined time interval Δ*t*. After having determined the actual current density in that time interval the bias voltage is then adjusted for the next time interval in a repeating feedback loop. Here, all disorder configurations are made to run synchronously, such that the time-dependent current density can always be determined by averaging over all of them. Figure 5 shows for a typical device how the device-integrated radiative decay rate [panel (A)], the current density [panel (B)] and the voltage [panel (C)] vary with time. The voltage shows a short overshoot early in the equilibration phase due to the feedback loop. A first vertical dashed line indicates roughly the equilibration time *t*_eq_, and a second vertical dashed line indicates the simulated time *t*_0_ at which the degradation processes are switched on. The statistical accuracy with which the time dependent decay and voltage change are obtained may then be estimated in the following way. As will be shown in Section 3.3 the typical simulated time at which a 50% reduction of the radiative decay rate has been reached is *t*_sim_ = 100 µs. A simulation for one 50 × 50 nm^2^ device (area *A*), carried out during this time period at a current density *J*_0_ = 200 Am^−2^ (close to the actual value at 9 V, see Figure 2A), would lead in the absence of efficiency loss processes to about *J*_0_ *t*_sim_ *A*/*q* ∼ 300 radiative decay events. Here *q* is the elementary charge. The actual time-integrated decay will be somewhat less due to the degradation. By averaging over 32 simulation boxes as mentioned in Section 2.1 we then obtain a fair a statistical accuracy for the time-dependence of the radiative decay rates and the voltage shift.

There is obviously a huge difference between the relevant timescales for device degradation, with lifetimes of hundreds of hours or more, and the electronic and photophysical processes in the picosecond to microsecond range. The huge discrepancy between these time scales shows already that the probability *P*_deg_ with which photophysical process trigger a degradation event must be very small. In Giebink et al. (2008), *P*_deg_ is estimated to be of the order of 10^–9^ from experiments of exciton-polaron quenching in small-molecule OLEDs. KMC OLED lifetime simulations that use the actual values of *P*_deg_ are therefore practically infeasible. However, it is possible to make the simulations feasible without a loss of the predictive quality, viz. by carrying them out for a strongly enhanced value of *P*_deg_ (Coehoorn et al., 2015). That is possible because the process of degradation is, by many orders of magnitude, the slowest process in the system. If *P*_deg_ is taken to be the same for all triggering processes and on all materials, the overall degradation rate is directly proportional to *P*_deg_. In this work, we simulate the degradation in the fastest possible manner, with *P*_deg,sim_ = 1. We find that for the devices that are studied, this leads to an LT50 lifetime that in almost all cases considered well exceeds the radiative decay rate. Even though that suggests that *P*_deg,sim_ = 1 is permitted, we plan to investigate the sensitivity to *P*_deg,sim_ in future work.

After the simulation, a comparison between the experimental lifetime, *τ*_exp_, and the simulated lifetime, *τ*_sim_, both obtained for the same current density, can be used to obtain the effective value of *P*_deg_ using the expression

$$P_{\deg}=P_{\deg,sim}\times \frac{\tau_{\text{sim}}}{\tau_{\exp}}.$$ (1)

In general, *P*_deg_ can be expected to vary for different materials and for different degrading processes, and to show a distribution instead of being single-valued. At present, no experimental study revealing the precise nature of the molecular scale degradation processes that occur in OLEDs is available. Furthermore, also no realistic estimates are available of values of *P*_deg_ that could more generally be expected for certain triggering processes and for certain materials. We can therefore presently not expect that KMC simulations can predict OLED lifetimes. However, the simulations might be expected to give a realistic estimate of effective values of *P*_deg_, and are expected to be able to predict trends in lifetime when the device architecture (such as the layer thicknesses) or operational conditions (such as the current density) are changed.

#### 3.1.3 Accelerated Lifetime Simulations

Just as often done experimentally, the simulations can be performed at increased current densities *J*_acc_ to achieve an accelerated degradation rate and to estimate by extrapolation the degradation rate at smaller current densities (Swayamprabha et al., 2020). That can be useful if, even when taking *P*_deg,sim_ = 1, the simulations would take unreasonable amounts of time. In experiments, the operational lifetime *τ*(*J*), is defined as the time after which the luminance has dropped to a certain fraction of the initial value, at a constant current density *J*. The dependence of *τ*(*J*) on the current density is often found to be well approximated by a power law: *τ*(*J*) ∝ *J* ^−*n*^. A determination of the exponent *n*, from experiment or from simulations at various current densities, then allows predicting the lifetime under accelerated conditions using

$$\tau \left(J\right)={\left(\frac{J_{\text{acc}}}{J}\right)}^{\;n}\tau \left(J_{\text{acc}}\right)\equiv \alpha_{J}\tau \left(J_{\text{acc}}\right).$$ (2)

Here *α*_*J*_ is the rescaling factor. The lifetime acceleration exponent *n* is often found to be in the range 1–2. For devices with a uniform emission profile, *n* was predicted to vary in the range 1.5–2, depending on the effective charge carrier density dependence of the mobility in the emissive layer due to energetic disorder or due to trapping of charge carriers on guest molecules in the host-guest system (Coehoorn et al., 2015). The shape of the emission profile at different voltages strongly impacts both the roll-off as well as the acceleration exponent. Smaller or larger values of *n* are expected when a given emission profile becomes more uniform or less uniform, respectively, as a result of the application of a higher voltage. The precise value of *n* can be determined by a superposition of various degradation mechanisms (Bangsund et al., 2018), and can depend on the lifetime definition used, for example LT50 or LT90.

### 3.2 Degradation Scenarios

We consider two types of degradation triggering events in this work: exciton-polaron quenching and exciton-exciton annihilation. These bimolecular processes give rise to a high local energy density and can therefore be expected to be most likely to result in degradation. This is consistent with the observation that balanced charge carrier and exciton profiles, which reduce the rate of these density-dependent bimolecular processes, are optimal for long lifetimes (Sim et al., 2020). No distinction will be made concerning the type of exciton that is involved in such processes (singlet or triplet), or whether the process was Dexter or Förster mediated. In all scenarios, we take the degraded materials to have no radiative decay, and only a non-radiative singlet and triplet decay rate of *k*_nr,S_ = 10^–8^ s^−1^ and *k*_nr,T_ = 10^–4^ s^−1^, respectively. This change is most impactful for the emitter sites, on which nearly all radiative decay takes place. We thus consider the assumption that a degraded emitter site can no longer contribute to the light emission and effectively acts as a fast exciton quenching site, as the fundamental reason for the luminance decay over time.

**FIGURE 6:**
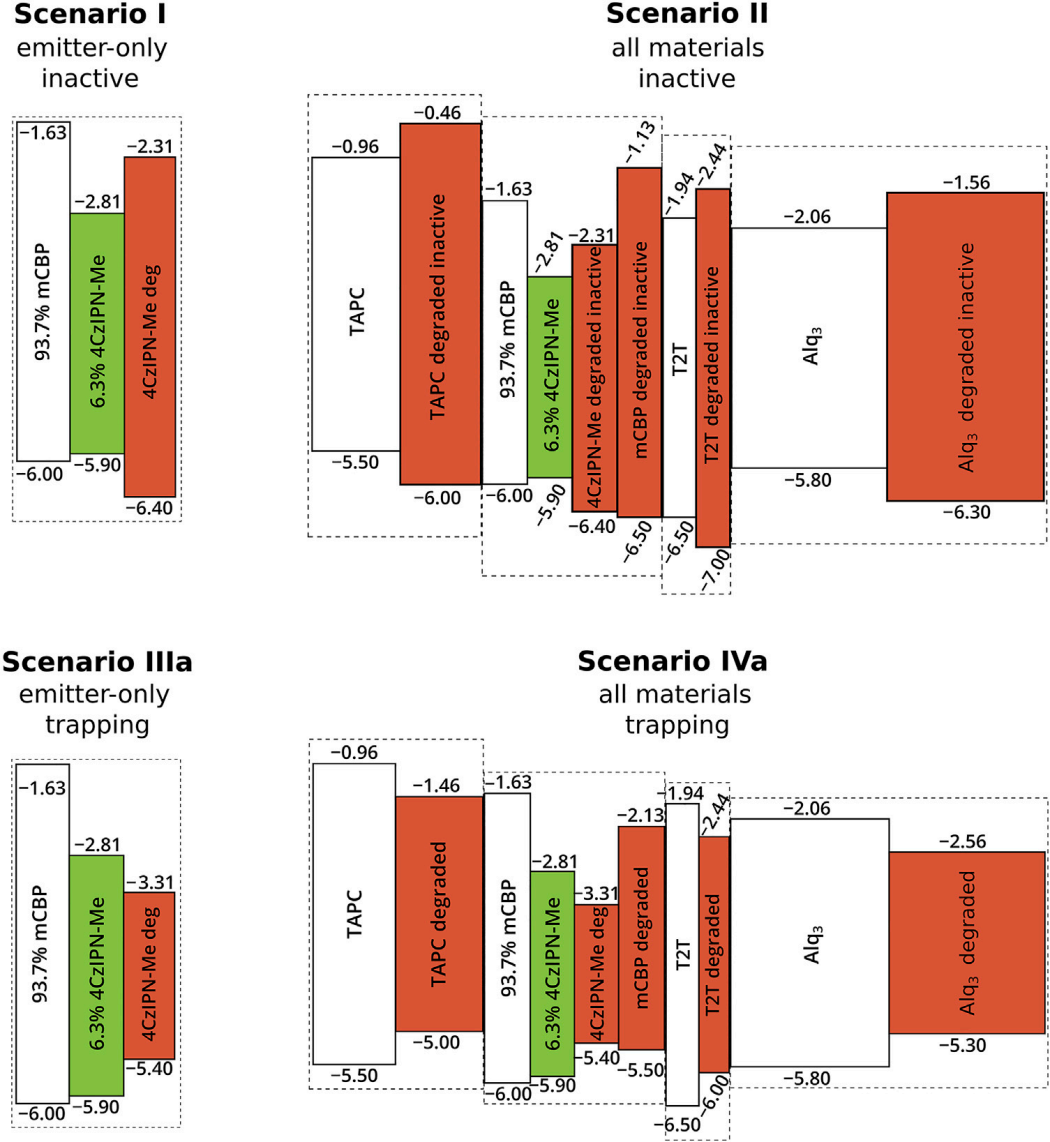
Overview of the simulated degradation product scenarios I, II, IIIa and IVa. The undegraded materials are shown in white (green for the emitter) and degradation products are red. In scenarios I and II, the degradation process results in a symmetric *increase* by 1 eV of the HOMO–LUMO gap of the degrading material, while scenarios IIIa and IVa lead to a symmetric *decrease* of that gap by 1 eV. In scenarios IIIb and IVb (not shown), degradation leads to a symmetric decrease of the gap by 2 eV. In scenarios I and III, only degradation the TADF emitter material 4CzIPN-Me is considered, while in scenarios II and IV all materials in the stack can degrade.

In our study, we distinguish six scenarios that differ concerning the degradation product and concerning the materials that are allowed to degrade. Within scenarios I and II, the degradation process results in a symmetric *increase* by 1 eV of the HOMO–LUMO gap of the degrading material, making it energetically unfavorable to be occupied by a charge with respect to the undegraded material. In contrast, Scenarios III(a,b) and IV(a,b), lead to a symmetric *decrease* of the energy gap by 1) 1 eV or 2) 2 eV. The result is that the degraded molecules act as an electron and hole trap relative to their undegraded state, with an average trap depth of 1) 0.5 eV or 2) 1.0 eV. In scenarios I and III(a,b), only degradation of the TADF emitter material 4CzIPN-Me is considered, while in scenarios II and IV(a,b) all materials in the stack can degrade. Figure 6 visualizes the changes to the frontier orbital energies for scenarios I, II, IIIa and IVa.

In all scenarios, the singlet and triplet energies of the degraded material are adjusted to the changes of the HOMO–LUMO gap, while the exciton binding energies are assumed to stay constant. As a result, a degradation product that is energetically unfavorable for charge transport (“inactive site”, as in scenarios I–II) is also energetically unfavorable for exciton formation and diffusion. On the other hand, a degradation product that is charge-trapping (scenarios III–IV) is also exciton-trapping.

### 3.3 Simulation Results

Lifetime simulations for the experimentally selected current density of 19 Am^−2^, leading to an initial emission of 1,000 cdm^−2^ in experiment (Furukawa et al., 2015), would take a significant amount of simulation time,^3^ even when using *P*_deg,sim_ = 1. We therefore carried out an extrapolation from simulations at larger current densities, using the approach that was outlined in Section 3.1.3. For the results in this section we perform the simulations at *J*_0_ = 190 Am^−2^. Simulation results from which the lifetime acceleration exponent *n* is determined are presented in Section 3.3.2. This is used in Section 3.3.3, where the simulation results are compared with experiment, leading for each scenario to an estimation for the degradation probability parameter *P*_deg_.

#### 3.3.1 Luminance Decay and Voltage Shift

**FIGURE 7:**
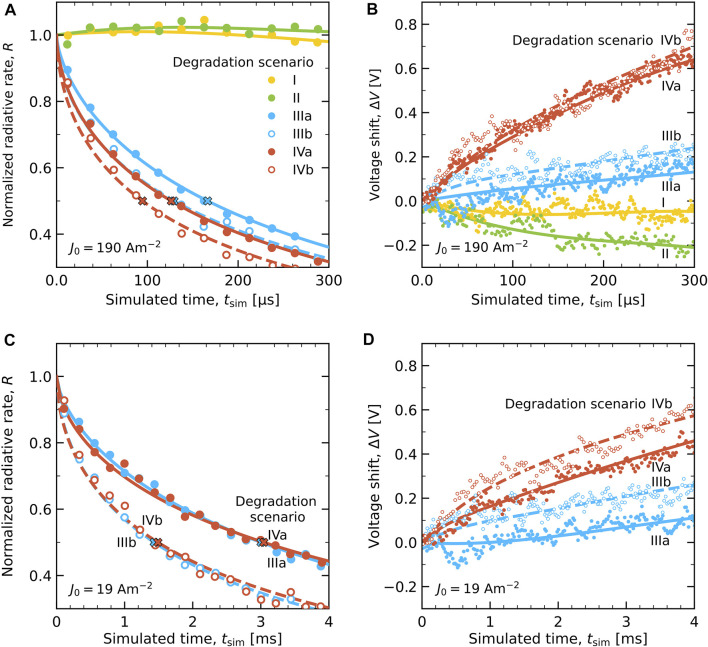
Degradation simulation results with full symbols for scenarios I, II, IIIa and IVa, and hollow symbols for IIIb and IVb. **(A)** The normalized radiative rate *R*(*t*) of all six scenarios at *J*_0_ = 190 Am^−2^ with crosses marking the LT50 lifetimes, *τ*_50,sim_. The curves are stretched exponential fits for scenarios IIIa and IVa (dashed curves for IIIb and IVb). **(B)** The voltage shift Δ*V* of all six scenarios with respect to the equilibrated pristine state (*t* = 0) at *J*_0_ = 190 Am^−2^. **(C)** *R*(*t*) after rescaling to *J*_0_ = 19 Am^−2^ by applying the scenario-specific current-rescaling factor *α*_*J*_ (see Table 2) for scenarios III(a,b) and IV(a,b). **(D)** Corresponding Δ*V* of scenarios III(a,b) and IV(a,b) at *J*_0_ = 19 Am^−2^.

Figure 7 shows the results of lifetime simulations for all six degradation scenarios I–IVb at the accelerated current conditions of *J*_0_ = 190 Am^−2^, using *P*_deg,sim_ = 1. The hollow symbols indicate the scenarios with deep traps from degradation (IIIb and IVb). Figure 7A shows the decay of the radiative rate *R*(*t*), normalized to the value in the equilibrated pristine device state. The overall radiative decay rate *R*(*t*) is taken to be proportional to the luminance decay, *L*(*t*). We thus neglect a possible effect on *L*(*t*) of a possible spectral shift. We remark that the radiative decay occurs almost exclusively on the TADF emitter. The figure shows that for scenarios I and II the radiative rate does not decrease with time, even though the rate of degradation is similar (and initially precisely equal) to that for the other scenarios. This indicates that when the degradation product is taken to be “inactive”, the excitons are easily generated on other, non-degraded emitter sites, and can contribute to the emission from those sites. We therefore regard these two “inactive site” degradation scenarios as inapplicable to this device.

For scenarios III(a,b) and IV(a,b), for which degradation results in charge and exciton-trapping sites, the simulations show a strong decay of the radiative rate within the simulated timescale. The crosses in Figure 7A indicate for each case the LT50 values, *τ*_50,sim_. The simulations show for scenarios III(a,b), for which degradation is limited to the emitter, a somewhat longer lifetime than for scenarios IV(a,b), for which all materials can degrade. However, the difference in lifetime for the two most extreme scenarios (IIIa versus IVb) is only approximately a factor 2. The numerical values of *τ*_50,sim_ at *J*_0_ = 190 Am^−2^ are included in Table 2, which gives an overview of all parameters extracted from the lifetime simulation results for the different scenarios.

**TABLE 2:** Results and analysis of degradation simulations for scenarios III(a,b) and IV(a,b) at accelerated current conditions (*J*_acc_ = 190 Am^−2^) and *P*_deg,sim_ = 1. The simulations yield a lifetime *τ*_50,sim_(*J*_acc_) and a voltage increase Δ*V* at *t* = *τ*_50,sim_(*J*_acc_). Extrapolation using the calculated acceleration exponent *n* (see Section 3.3.2), yielding an acceleration factor *α*_*J*_ (see Eq. 2), leads to *τ*_50,sim_(*J*_0_), with *J*_0_ = 19 Am^−2^ the current density at which an experimental lifetime of 1,470 h has been obtained (Furukawa et al., 2015). With Eq. 1, the simulated and experimental lifetimes at *J*_0_ are used to calculate the effective degradation probability per triggering event, *P*_deg_.

Scenario	*τ*_50,sim_(190 Am^−2^)[µs]	Δ*V*(190 Am^−2^)[V]	*n*	*α*_*J*_	*τ*_50,sim_(19 Am^−2^)[ms]	*P*_deg_ × 10^9^
IIIa	166	0.09	1.26	18.0	3.00	0.57 ± 0.11
IIIb	129	0.15	1.04	11.1	1.43	0.27 ± 0.07
IVa	126	0.36	1.38	24.1	3.04	0.58 ± 0.14
IVb	95	0.32	1.20	15.7	1.49	0.28 ± 0.10

Figure 7B shows the simulated voltage shift Δ*V* with respect to the value of ∼9.8 V in the equilibrated pristine state (see Figure 2A). Just as for the radiative rate, scenarios I and II with inactive degradation products do not show the experimentally observed degradation behaviour: the simulations do not predict an increase of the driving voltage with time. For scenario II, even a shift to lower voltages is observed. This could be a result of exciton-polaron quenching involving low-energy sites in the density of states, where charges can be trapped even in the pristine device. Degradation scenarios that make such sites less trapping (like I and II) lead to a density of states that is effectively narrowed, resulting in easier charge transport. Also on the basis of these findings, we regard the “inactive site” degradation scenarios as inapplicable to this OLED. On the other hand, the simulations for scenarios III(a,b) and IV(a,b) show an increase of the required driving voltage. The numerical values of Δ*V* at *J*_0_ = 190 Am^−2^, evaluated at *t* = *τ*_50,sim_, are included in Table 2. Interestingly, the effect is relatively insensitive to the precise value of the assumed average trap depth of the degradation product (0.5 or 1.0 eV), but quite sensitive to whether only the emitter molecules or all molecules can degrade. A comparison with the bimolecular loss rate profiles in Section 2.2 indicates that the increased voltage shift is a result of degradation caused by exciton-polaron quenching at the HTL∣EML and EML∣HBL interfaces.

#### 3.3.2 Determination of the Acceleration Exponent

**FIGURE 8:**
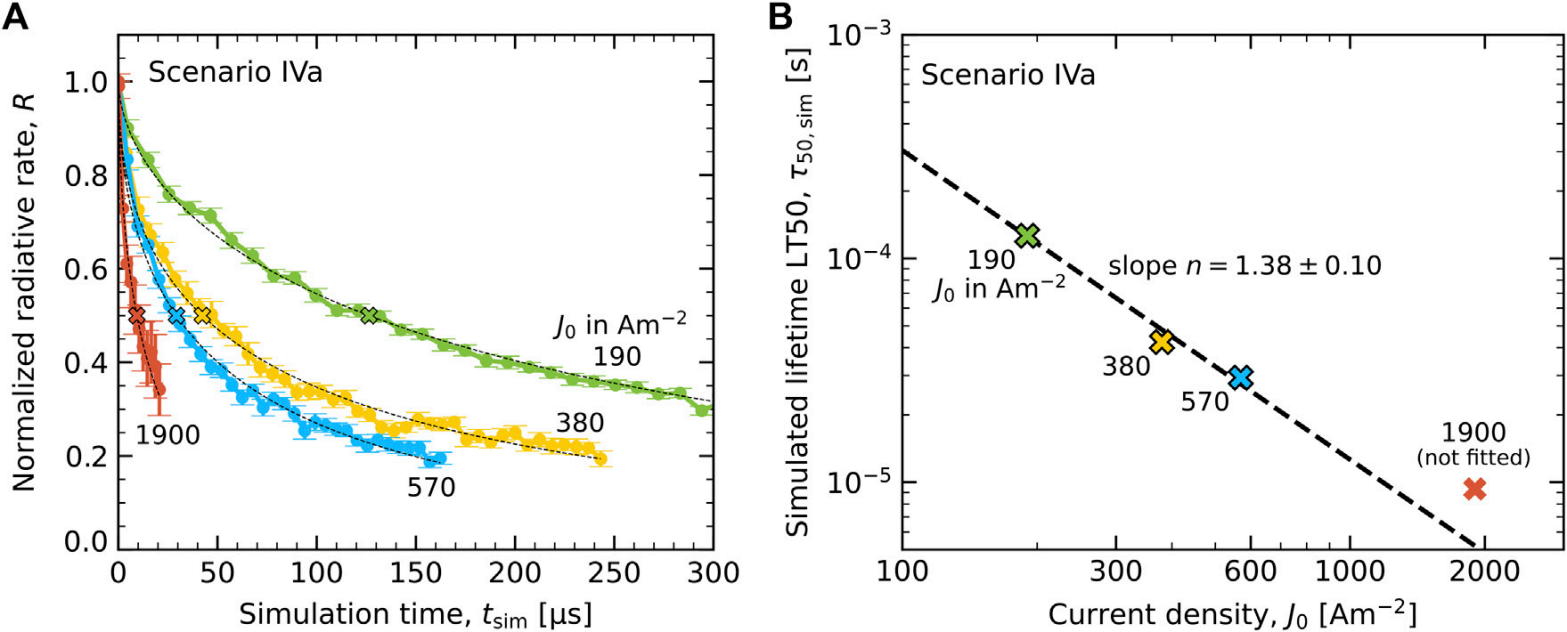
Determination of the acceleration exponent (see Section 3.1.3) for scenario IVa. **(A)** Simulated radiative decay rate *R*(*t*) at four values of the current density, normalized to the equilibrated value of *R* in the pristine state. Dashed curves give stretched exponential fits to the simulation results. Crosses mark the LT50 lifetimes *τ*_50,sim_(*J*_0_). **(B)** Current density dependence of *τ*_50,sim_(*J*_0_). The dashed line is a linear fit through the three lowest current density points with a slope *n* = 1.38 ± 0.10.

We have for scenarios III(a,b) and IV(a,b) determined the acceleration exponent *n*, using the approach that was outlined in Section 3.1.3, in order to enable making a comparison with the experimental lifetime study for the current density of 19 Am^−2^ that was used by Furukawa et al. (2015). As an example, Figure 8A shows for degradation scenario IVa the simulated decrease of the normalized radiative decay rate, *R*(*t*), obtained from constant current simulations and normalized to the equilibrated pristine state value for each current density. Figure 8B shows the resulting current density dependence of the simulated LT50 lifetime, *τ*_50,sim_, determined from stretched exponential fits to the simulation curves in panel (A), which are indicated by thin dashed lines. The uncertainty is approximately equal to the symbol size. The figure shows that the decrease is non-linear on a double-log scale towards higher current densities, and hence does not precisely follow a power law. A linear fit through the three lowest current density data points yields an acceleration exponent of *n* = 1.38 ± 0.10. The observed non-linear decrease on the log-log scale may be indicative of a gradually changing degradation mechanism, e.g., due to shifting charge carrier and exciton density profiles. It should be noted that for *J*_0_ = 1900 Am^−2^, the largest current density considered, *τ*_50,sim_ is approx. 10 µs, which is only one order of magnitude larger than the radiative decay time. The acceleration exponents for scenarios IIIa, IIIb and IVb have been determined in a similar way (see Supplementary Figure S3 in the SM), and are included in Table 2.

#### 3.3.3 Comparison With Experiment

A comparison of the simulations results with the experimental results that are reported in Furukawa et al. (2015) can be made by first extrapolating the simulation result to the experimentally used current density of *J* = 19 Am^−2^ and by subsequently comparing the shapes of the simulated and experimental radiative decay and voltage shift curves. The necessary parameters are summarized in Table 2 for scenarios III(a,b) and IV(a,b). The last column gives the values of *P*_deg_ that follow from Eq. 1 with the simulated *τ*_50_ lifetime, obtained assuming *P*_deg,sim_ = 1, and the experimental lifetime. For scenario IVa, e.g., *τ*_50,sim_(19 Am^−2^) is obtained from *τ*_50,sim_(190 Am^−2^) using a scaling factor *α*_*J*_ = (190/19)^*n*^ = 24.1. A comparison of the simulated LT50 lifetime and the experimental value (1,470 h) leads then with Eq. 1 to *P*_deg_ = 0.58 × 10^–9^.

The simulation results after the current rescaling are shown in Figure 7. Figure 7C shows the decay of the radiative rate for each scenario. The *R*(*T*) curves for the scenarios with a trap depth of 0.5 eV (IIIa and IVa), and as a result also the values of *τ*_50,sim_(19 Am^−2^) and *P*_deg_, are very similar. For the scenarios with a trap depth of 1 eV (IIIb and IVb), *τ*_50,sim_(19 Am^−2^) and *P*_deg_ are approximately a factor two smaller. For the scenarios studied here, *P*_deg_ and the decay of *R*(*t*) at *J*_0_ = 19 Am^−2^ are found not to be dependent on the degradation of non-emitting materials, but do show a sensitivity to the average trap depth after the degradation. Extending the analysis to obtain more specific information, e.g., about the dependence of *P*_deg_ on the type of bimolecular process is beyond the scope of this work. We remark that the values of *P*_deg_ that we obtain here for a green TADF OLED are slightly smaller than the value of *P*_deg_ ≈ 2 × 10^–9^ that was estimated from experiments by Giebink et al. (2008) for exciton-polaron quenching in a green phosphorescent OLED. Figure 7D shows the voltage shift for the four scenarios after the lifetime rescaling to lower current-densities. Similar as under the accelerated current density conditions in panel (B), the increase of Δ*V* is significantly stronger if the non-emitting materials contribute to the degradation process (IVa and IVb).

**FIGURE 9:**
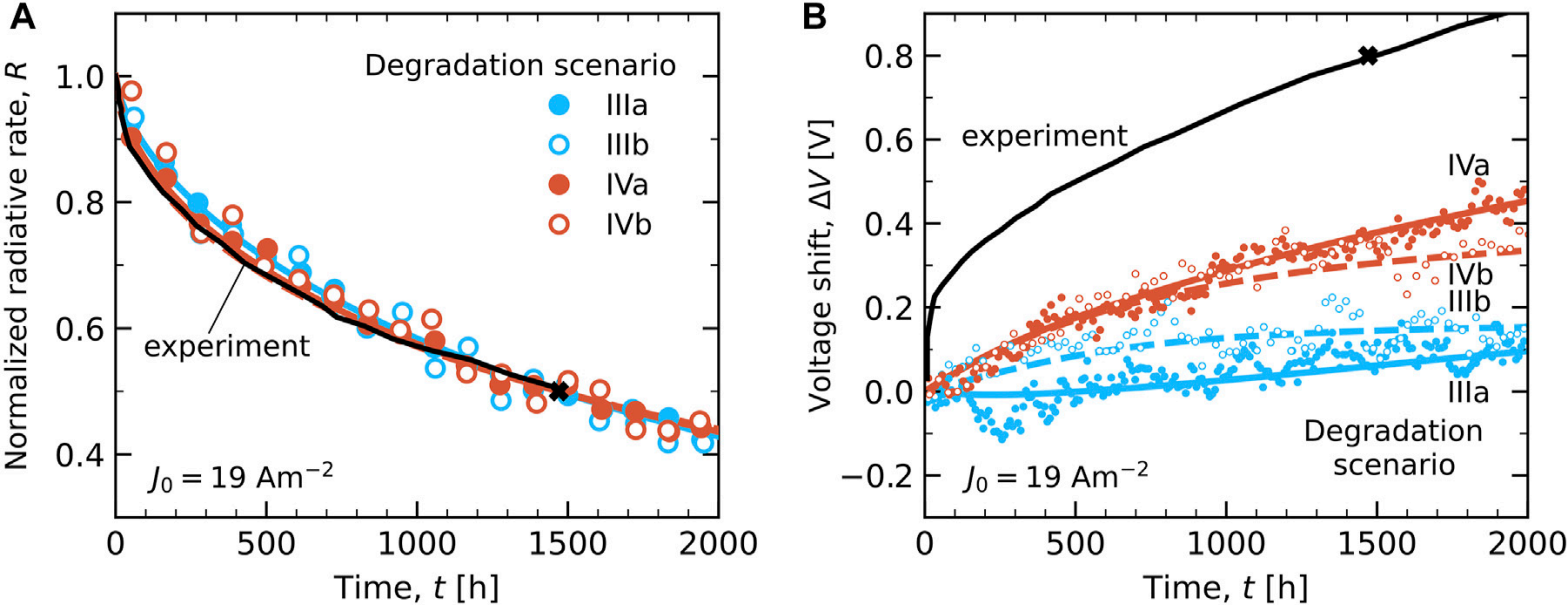
Comparison of the experimental (black curves) and simulation results (symbols) at the same current density *J*_0_ = 19 Am^−2^ for degradation scenarios III(a,b) and IV(a,b). Full symbols, and curves as guides-to-the-eye, are used for scenarios IIIa and IVa, and hollow symbols, with dashed curves, are used for scenarios IIIb and IVb. The black cross gives the experimental LT50 lifetime (1,470 h). **(A)** Simulated radiative decay rate *R*(*t*), normalized to the equilibrated value in the pristine state (*t* = 0). **(B)** Voltage shift Δ*V* with respect to the equilibrated pristine state.

Figure 9 gives a comparison of the experimental data from Furukawa et al. (2015) (black curves) and the simulation results. Panel (A) compares for each simulation scenario the decay of the radiative rate, normalized to the value in the equilibrated pristine state and scaled using the value of *P*_deg_ that is given in Table 2, with the normalized experimental decay of the luminance. For each scenario, the time-dependence of the decay is very similar to the experimental curve. This comparison thus does not yet enable us to identify a most likely degradation scenario. Figure 9B shows the experimental and simulated voltage shift Δ*V* for the four scenarios. The experimental voltage shift of 0.8 V at *t* = *τ*_50, exp_ is even for scenario IV, which includes degradation and trap site formation in all layers, significantly underestimated. The figure shows that the experimental voltage increase is particularly fast in a very early stage of the degradation process. Our simulations suggest that this effect is not a result of intrinsic processes in the active part of the device. Also in other work, extrinsic factors have been argued to strongly affect the early-stage luminance (“fast-initial drop”) (Yamamoto et al., 2011) and the voltage shift (Sim et al., 2020). We can also not exclude that the observed fast initial increase of the voltage shift is related to degradation processes in the hole-injecting layer, at the interface of that layer with the TAPC hole transport layer, or at the electron-injection layer, which are not explicitly included here.

#### 3.3.4 Degradation Profile

**FIGURE 10:**
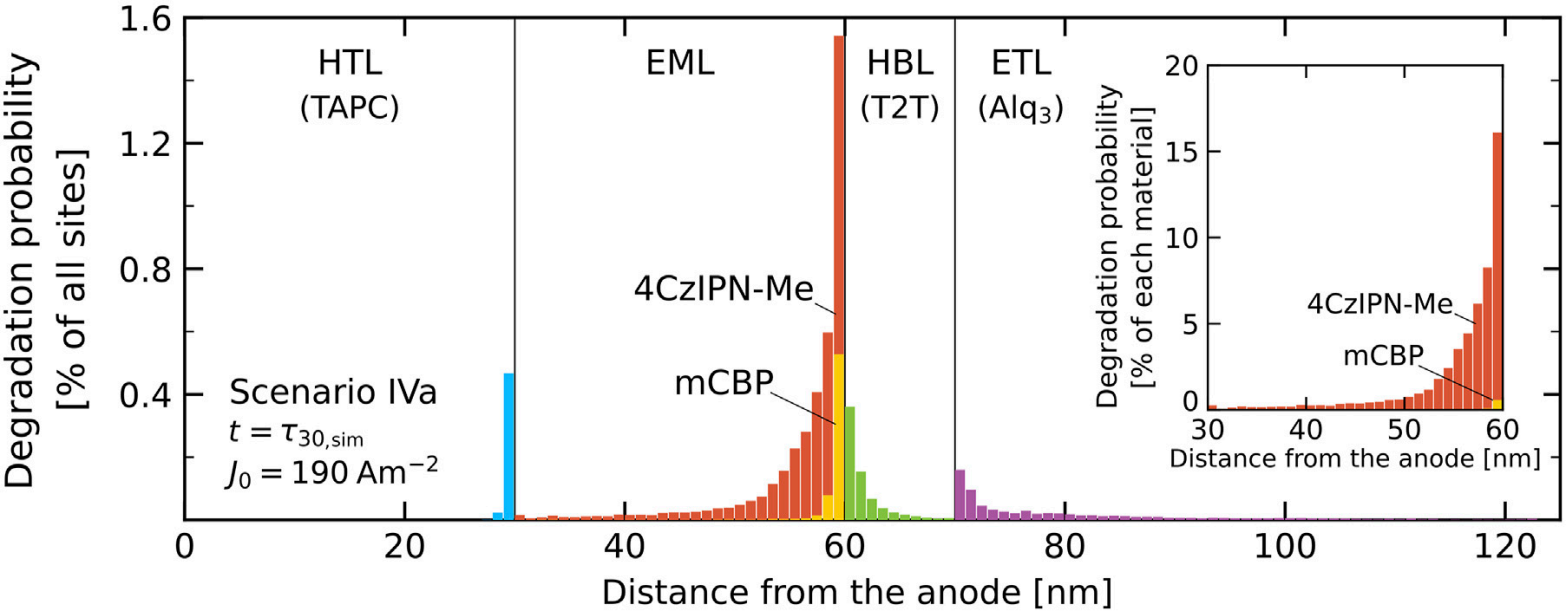
Degradation profile, defined as the percentage of degraded sites in each molecular layer, for scenario IVa and *J*_0_ = 190 Am^−2^ at the LT30 lifetime (*τ*_30_). For the EML the contributions of the host and guest molecules are added, so that the total bar height corresponds to the total percentage of degraded sites. The inset shows the degradation in the EML for both materials as a percentage of the number of sites of that material.

The simulations can provide the layer or even site-resolved probability that a degradation process has occurred. As an example, Figure 10 shows the degradation profile that has been obtained for scenario IVa for *J*_0_ = 190 Am^−2^ at a simulated time *t*_sim_ = 381 µs, corresponding to the LT30 lifetime, *τ*_30,sim_. Within this scenario, degradation can occur on all materials, in all layers. The figure shows that in the EML most degradation occurs near the interface with the HBL, and that in the molecular layer adjacent to that interface ∼1.5% of the molecules have degraded. The dominating degradation-triggering process is exciton-polaron quenching of singlet excitons located on an emitter site by electrons that are located on another nearby emitter site. Even though the average electron density in the EML is approximately thirty times larger than the layer-averaged hole density, the contribution of exciton-electron quenching is found be only a factor 7 larger than the contribution of exciton-hole quenching. This is because in the EML the diffusivity of the holes, which can also hop via the host molecules, is much larger than that of the electrons. As a result, about one third of the degraded molecules in this layer is an mCBP host molecule. The inset shows that due to the difference in concentrations the probability that an emitter is degraded is much larger than the probability that a host molecule is degraded. The degradation of host molecules in the EML can still contribute to the luminance loss, because these degraded molecules act as trap sites on which an electron or hole can reside that gives rise to exciton-polaron quenching. Similarly, also degraded molecules in the HBL near the interface with the EML can give rise to such quenching. That can explain why for scenario IVa (IVb) the simulated lifetime at high current densities is about 25–30% shorter than for scenario IIIa (IIIb). The degradation of sites in the HTL near the interface with the EML is expected to contribute only weakly to the luminance decay, because of the small exciton density near that interface (see Figures 3C,D). However, the trapping of charges at these sites will contribute to the voltage shift.

## 4 Summary and Conclusion

We have demonstrated how kinetic Monte Carlo OLED device simulations can be used to investigate the sensitivity to various degradation scenarios. Within a degradation scenario, events are defined that can trigger a molecular degradation process, the probability *P*_deg_ with which such an event triggers degradation, and the final product of each degradation process. The simulations have been applied to an experimentally well-characterized green TADF OLED (Furukawa et al., 2015). Bimolecular loss-processes (exciton-polaron quenching and exciton-exciton annihilation) are assumed to be the degradation-triggering processes, and the degradation product is assumed to be a non-emissive molecule. The scenarios that are included differ with respect to the energy of the frontier orbitals of the degraded molecule and the type of molecules considered to be degrading (only the TADF emitters, or also the other materials). The probability *P*_deg_ that a triggering event gives rise to a degradation process is expected to be process- and material-specific, and could be given by a distribution due to the molecular scale disorder. We have neglected such complications in this paper by treating *P*_deg_ as a single effective parameter that can be deduced for each scenario from a comparison between the simulated and experimental luminance decay for a fixed certain current density. The degradation simulations are carried out after having obtained an equilibrated charge and exciton density at the selected current density and after having determined the device performance in its pristine state with a statistically good accuracy. Subsequently, the possibility of degradation is enabled in the simulation.

In order to make explicitly simulating the entire device lifetime simulations feasible, the value of *P*_deg_ that is used in the simulations is taken as large as possible, with the constraint that the degradation process is still slower than the radiative and non-radiative decay processes. For the devices studied, we find that degradation simulations for the experimentally used current density (∼20 Am^−2^) still take too long. We therefore determine the simulated lifetime by extrapolation of results from accelerated degradation simulations at larger current densities. Such a procedure is also often used experimentally. For the selected TADF OLEDs, the simulations show that scenarios for which the HOMO–LUMO gap of the degraded materials is increased relative to their undegraded state lead to essentially no luminance decay and almost no voltage shift or even a small negative shift. In contrast, scenarios in which the degraded molecules form trap sites of various depth are found to provide a time-dependence of the luminance decay that agrees well with experiment. For all trapping scenarios, the experimental results can be quite well described using *P*_deg_ ∼ (0.2–0.7) × 10^–9^. The predicted voltage shift is largest when degradation of all materials is assumed, but is significantly smaller than the actually observed shift. The difference is indicative either of the role of degradation processes that involve extrinsic factors or degradation at or near the injecting contact layers.

We envisage that the methodology that has been demonstrated in this work can be used to systematically investigate how OLED lifetimes depend on the layer composition and structure and the charge carrier balance. A wide range of potentially interesting additional degradation scenarios could be used to study the sensitivity of the luminance decay or voltage increase to degradation in spatially distinct parts of the device, for example by limiting the degradation to specific materials, specific layers, or even to smaller regions within a certain layer. Furthermore, experiments (Kondakov et al., 2007; Sandanayaka et al., 2015) or quantum-chemical calculations (Hong et al., 2016; Wang et al., 2021) that predict the probability of degradation-triggering events in each material and the properties of the resulting degradation products could be used to narrow the range of relevant degradation scenarios. A slightly different application of KMC lifetime simulations is to study systematically how the lifetime acceleration exponent *n*, which is also an important experimental parameter, is related to the details of the OLED device structure and the assumed degradation processes. A next step would be to refine the approach that has been presented in this work, so that material- and process-specific instead of device-averaged effective values of *P*_deg_ are obtained. Such refined results could then be correlated with the results of first principles quantum-chemical modelling.

## Data Availability

The raw data supporting the conclusions of this article will be made available by the authors, without undue reservation.

## Footnotes

1 The exciton binding energy of 4CzIPN-Me is assumed to be equal to that of 4CzIPN, studied in Yoshida and Yoshizaki (2015).

2 The Bumblebee software is provided by Simbeyond B.V.; https://simbeyond.com/

3 The degradation phase of the simulations at J_0_ = 190 Am^2^ takes 24–72 h to reach the LT50 lifetime. A simulation at 19 Am^2^ is expected to take approximately a factor 10–25 longer (see α_J_ in Table 2).
