# Supplementary material for: Identification of OLED Degradation Scenarios by Kinetic Monte Carlo Simulations of Lifetime Experiments
Source: Front Chem. 2022 Jan 27;9:823210. doi: 10.3389/fchem.2021.823210 (PMC8828587; doi:10.3389/fchem.2021.823210)
Supplement: Supplementary file 1 [file DataSheet1.PDF]

Supplementary Material: Identification of OLED degradation scenarios by kinetic Monte Carlo simulations of lifetime experiments

Christoph Hauenstein [1,2,*], Stefano Gottardi [1], Engin Torun [1], Reinder Coehoorn [2,3] and Harm van Eersel [1]

[1] *Simbeyond B.V., Eindhoven, The Netherlands*
[2] *Department of Applied Physics, Eindhoven University of Technology, Eindhoven, The Netherlands*
[3] *Institute for Complex Molecular Systems, Eindhoven University of Technology, Eindhoven, The Netherlands*

Correspondence*:
Simbeyond B.V., Het Eeuwsel 57, 5612 AS Eindhoven, The Netherlands
christoph.hauenstein@simbeyond.com

S1 OVERVIEW OF SIMULATION PARAMETERS

For the device studied, the layer thicknesses, the layer compositions and the HOMO and LUMO energy levels of all materials have been given in Fig. 1 of the main text. Table 1 of the main text includes the energy levels, the radiative and non-radiative decay rates, and literature references. In Table S1, we present an overview of all other simulation parameters.

Table S1. Overview of the additional simulation parameters used.

Parameter	Description	Value
a	nearest-neighbor distance [1]	1 nm
ε_r	relative dielectric permittivity	3.0
σ	width (standard deviation) Gaussian HOMO and LUMO disorder [2]	0.10 eV
$\sigma_{\mathrm{S(T)}}$	width of the singlet (triplet) excitonic disorder [2]	0.05 eV
λ	wavefunction decay length (taken equal for electrons, holes and excitons)	0.3 nm
$\nu_{1,\mathrm{h}}$	nearest-neighbor hole hopping attempt rate [3 4]	1.66×10^{10} s^{-1}
$\nu_{1,\mathrm{e}}$	nearest-neighbor electron hopping attempt rate [3 4]	1.66×10^{9} s^{-1}
f_{S}	fraction of singlets formed upon exciton generation	0.25
$k_{\mathrm{ISC,4CzIPN\text{-}Me}}$	intersystem crossing rate on 4CzIPN-Me	41×10^{6} s^{-1}
$k_{\mathrm{rISC,4CzIPN\text{-}Me}}$	reverse intersystem crossing rate on 4CzIPN-Me	0.77×10^{6} s^{-1}
$R_{\mathrm{F,diff}}$	Förster radius for singlet exciton transfer[4 5 6 7]	1.5 nm
$k_{\mathrm{D,1}}$	nearest-neighbor Dexter transfer attempt rate [4 5 8]	2.1×10^{7} s^{-1}
$R_{\mathrm{F,Q}}$	Förster radius for singlet–polaron quenching [4 5 7]	3.5 nm
$R_{\mathrm{F,A}}$	Förster radius for singlet–exciton annihilation [4 5 7 9]	3.5 nm
T	temperature	300 K

[1] The sites reside on a simple cubic grid. The total site density is thus $N_{\mathrm{t}} = a^{-3} = 1 \times 10^{27}$ m^{-3}.
[2] The disorder is assumed to be spatially uncorrelated.
[3] Hops to neighbor sites within a $5 \times 5 \times 5$ nm cube around each site are included.
[4] The actual rate is thermally activated, as described by the Miller-Abrahams formula.
[5] Exciton transfer to or exciton interactions with neighbor sites within a $11 \times 11 \times 11$ nm cube around each site are included.
[6] The final spin state after Förster-transfer is in all cases assumed to be a singlet.
[7] The exciton transfer between all types of molecules was described using this value of R_{F}, except in the "emitter-only" Förster scenario.
[8] The process accounts for the spin statistics: S $+$ S $\rightarrow$ S, S $+$ T $\rightarrow$ T and T $+$ T $\rightarrow$ ¼S $+$ ¾T.
[9] For Förster-type SSA and STA the final state is a singlet and triplet exciton, respectively.

S2 SENSITIVITY TO THE CHARGE CARRIER HOPPING ATTEMPT RATE

As mentioned in the main text, the current-density for the initial (pristine) state that was obtained in our previous simulation study (see Ref. [2]) was a factor of two larger than the experimental value [1]. To address this, we have in the present study halved the hopping attempt rate for holes, $\nu_{1,\mathrm{h}}$, and electrons, $\nu_{1,\mathrm{e}}$. Fig. S1 shows the sensitivity of the $J(V)$ characteristics and the external quantum efficiency $\eta_{\mathrm{EQE}}(J)$ to this parameter change, assuming a light-outcoupling efficiency of 25%. The dashed curves are a guide-to-the-eye. In unipolar device simulations, a change of $\nu_{1,\mathrm{h}}$ or $\nu_{1,\mathrm{e}}$ by a certain factor would lead to a change of the current density at a given voltage by precisely the same factor. However, in bipolar device simulations there are several other rates that can also affect the current density, such as the rates of radiative decay, exciton dissociation and bimolecular excitonic processes. We find nevertheless that the current density is, within the accuracy of the simulations, precisely halved (panel (A)), and that the decrease of the EQE at higher current-densities ("roll-off") is nearly unchanged (panel (B)).

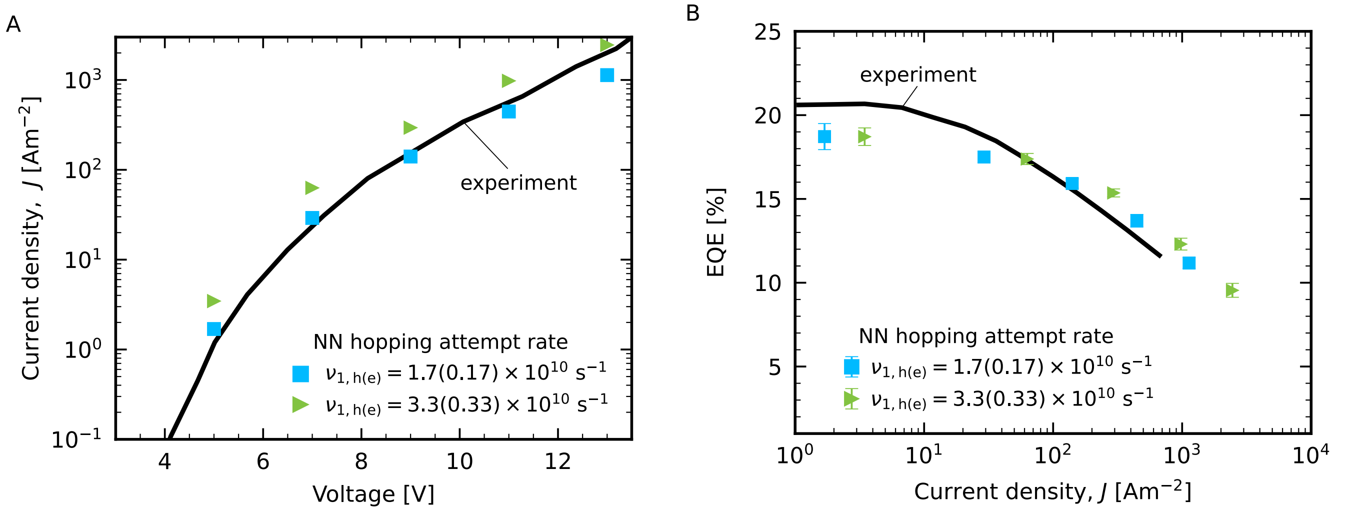

Figure S1. Simulation results revealing the sensitivity to a change of the hole and electron hopping attempt rates from the values used in Ref. [2] (green triangles) to the 50% reduced values that are used in this paper (blue squares). Black curves show the experimental results from Ref. [1]. (**A**) $J(V)$ characteristics and (**B**) EQE roll-off $\eta_{\mathrm{EQE}}(J)$ assuming a light out-coupling efficiency of 25%.

S3 VOLTAGE-DEPENDENCE OF THE EXCITONIC PROCESSES IN PRISTINE DEVICES – "EMITTER-ONLY" SCENARIO

Figure S2 shows the contribution of the various excitonic processes in the equilibrated pristine state for the "emitter-only" scenario, in which only Förster-type interactions between the emitter molecules are assumed. (see Sec. 2.1). Compared to the losses when Förster processes are enabled for all materials, shown in Fig. 4 of the main paper, this "emitter-only" scenario shows a smaller loss due to singlet-polaron quenching. Additionally, the contribution from singlet non-radiative decay is at high voltages slightly smaller.

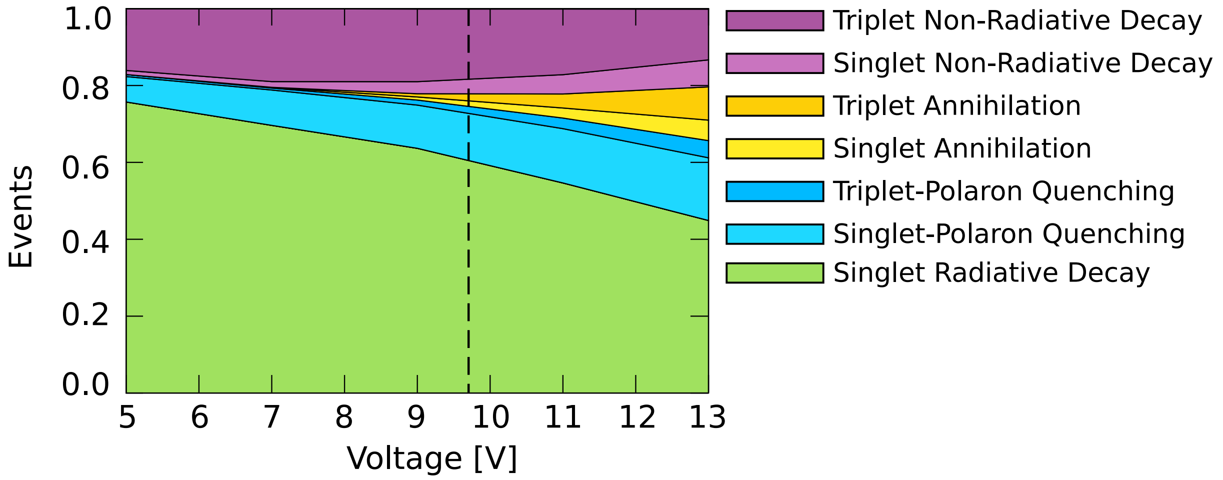

Figure S2. Relative contribution of the various excitonic processes for singlets and triplets for the case of Förster processes that occur only between sites of the emitter material. The dashed vertical line represents the voltage that corresponds to the current density $J_0 = 190\ \mathrm{Am}^{-2}$ at which the degradation simulations are performed.

S4 DETERMINATION OF THE ACCELERATION EXPONENT

In addition to the determination of the acceleration exponent n that was given for scenario IVa in Sec. 3.3.2 of the main paper, we give here the simulation results that have been used to determine n for the degradation scenarios IIIa, IIIb and IVb. Fig. S3 shows in panels (A, C, E) the simulated decrease of the radiative decay rate $R(t)$, obtained from constant-current simulations for various values of J_0 and normalized to the initial-state value for the selected current density. The crosses indicate the simulated LT50 lifetime, $\tau_{50,\mathrm{sim}}(J_0)$, determined from stretched exponential fits (dashed curves). Panels (B, D, F) show the resulting current density dependence of $\tau_{50,\mathrm{sim}}$ as a function of J_0. The data shows a linear relationship on a logarithmic scale. The absolute value of the slope gives the acceleration exponent n.

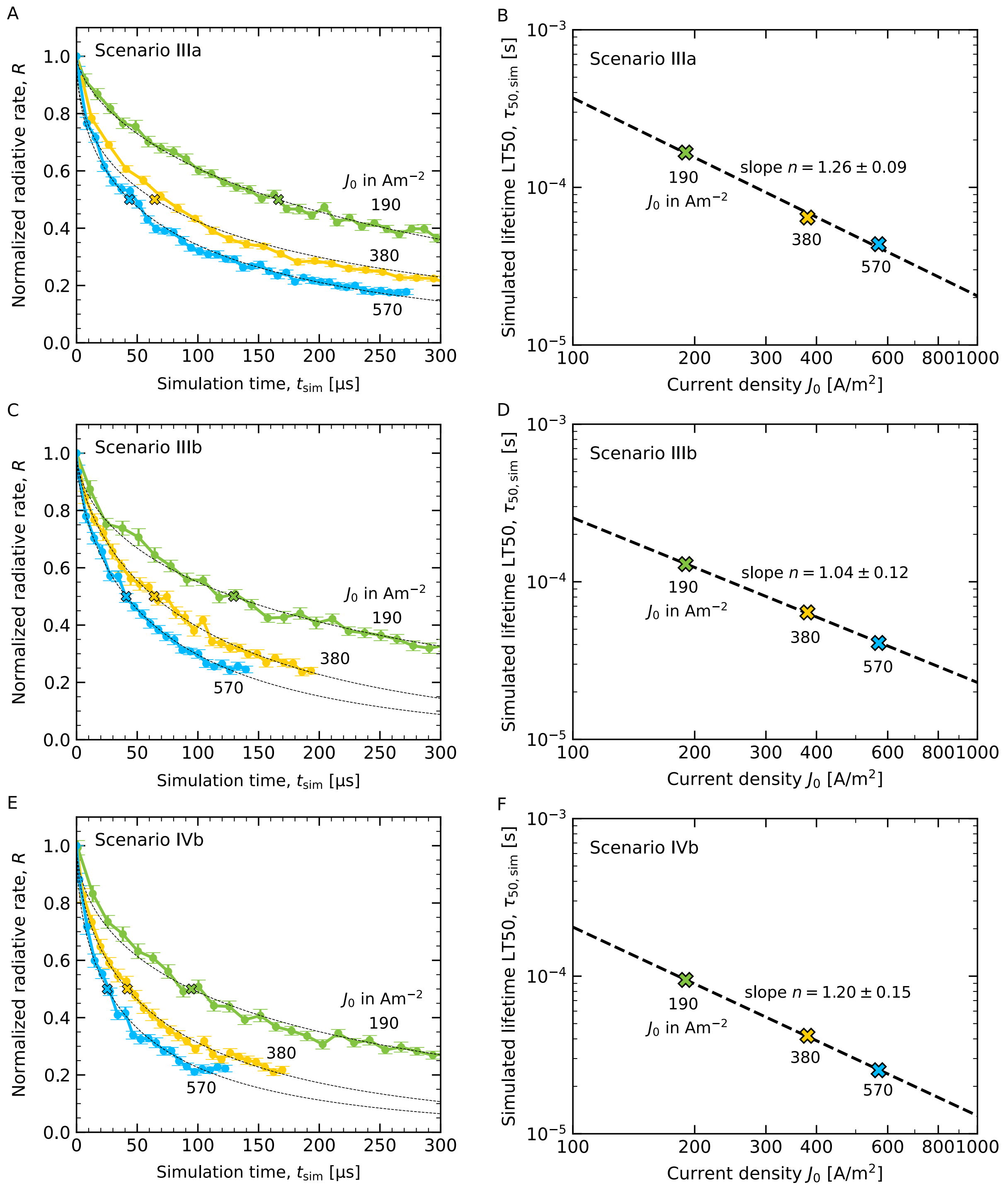

Figure S3. Determination of the acceleration exponent, n (see Sec. 3.1.3 of the main text), for scenarios IIIa, IIIb and IVb (see Sec. 3.2 and Fig. 6 of the main text). **(A, C, E)** Simulated decay of the radiative rate, $R(t)$, at varying current densities J_0, normalized to the initial-state value and using $P_{\text{deg,sim}} = 1$. Dashed curves give stretched-exponential fits to the simulation results. Crosses mark the LT50 lifetimes $\tau_{50,\text{sim}}(J_0)$, where the emission rate has decayed to half its initial value. **(B, D, F)** Current density dependence of $\tau_{50,\text{sim}}(J_0)$. The data shows a linear relationship on a logarithmic scale. The acceleration exponent n is equal to the absolute value of the slope.

REFERENCES

[1]T. Furukawa, H. Nakanotani, M. Inoue, and C. Adachi. Dual enhancement of electroluminescence efficiency and operational stability by rapid upconversion of triplet excitons in oleds. *Sci. Rep.*, 5:8429, 2015.

[2]S. Gottardi, M. Barbry, R. Coehoorn, and H. van Eersel. Efficiency loss processes in hyperfluorescent OLEDs: A kinetic Monte Carlo study. *Appl. Phys. Lett.*, 114(7):073301, 2019.
